# Single-cell RNA sequencing of shoot apex reveals the mechanism of cyclin regulating cell division via auxin signaling pathway in *Populus alba*

**DOI:** 10.3389/fpls.2025.1555388

**Published:** 2025-03-04

**Authors:** Jing-hui Liang, Zhao-qun Wu, Yue-Xuan Zhang, Ye-Bo Yang, Shi-Yi Wang, Meng-Yu Gai, Yu-Wen Wang, Xiu-Xing Zhang, Jing Xue, Bo-Hao Duan, Hai-Ling Yang

**Affiliations:** ^1^ State Key Laboratory of Tree Genetics and Breeding, National Engineering Research Center of Tree Breeding and Ecological Restoration, Beijing Forestry University, Beijing, China; ^2^ The Tree and Ornamental Plant Breeding and Biotechnology Laboratory of National Forestry and Grassland Administration, College of Biological Sciences and Technology, Beijing Forestry University, Beijing, China

**Keywords:** shoot apex, cyclin, cell division, auxin, ScRNA-seq, *Populus alba*

## Abstract

The shoot apex of *Populus alba* primarily comprises the shoot apical meristem, axillary meristem, leaf primordium, and young leaves, all of which exhibit high division potential. The single-cell RNA sequencing of the apical buds of *P. alba* can provide deeper insights into the processes of cell proliferation and differentiation, including the key genes and signaling pathways that regulate these processes. Scanning electron microscopy was used to examine the structure of the shoot apex, followed by single-cell sequencing analysis. A total of 29,011 cells were obtained from two biological replicates. Dimensionality reduction and clustering identified 17 distinct cell clusters. Pseudotime analysis revealed that shoot apex meristem cells and mesophyll cells emerged first, followed by the differentiation and maturation of vascular and intercalary meristem cells over time. Trichome differentiation occurred last, whereas epidermal cell differentiation persisted throughout development. At the single-cell level, auxin signaling pathway genes potentially involved in leaf tissue development were identified, along with an analysis of the expression specificity of *CYC* and *CDK* genes across mesophyll, epidermis, vascular, and shoot apex meristem tissues. These findings facilitate the elucidation of the molecular regulatory mechanisms by which *CYC* and *CDK* genes influence leaf development in *P. alba*.

## 1 Introduction

The shoot apex is characterized by a high degree of division potential and primarily comprises the shoot apical meristem (SAM), axillary meristem, leaf primordia, and young leaves (Zhang et al., 2021; Wang et al., 2018). During the development of plant apical buds, cell division and proliferation are essential to maintain meristem activity, allowing the apical bud to continuously generate new cells that support the vertical growth of the plant as well as the formation of lateral organs (Gaillochet et al., 2015; Wang et al., 2018). This process is complexly regulated by a variety of hormones, notably auxin and cytokinin, which interact through complex signaling networks to collaboratively shape the developmental pattern of the apical bud (Schuster et al., 2014; Zhao et al., 2010; Reinhardt et al., 2003).

Cyclins (CYCs) and cyclin-dependent kinases (CDKs) are crucial regulators of cell division, forming complexes that manage the various phases of the cell cycle, including the G1/S and G2/M checkpoints (Inzé and De Veylder, 2006; De Veylder et al., 2007). In *Arabidopsis thaliana*, at least 50 CYCs have been identified, categorized into groups A, B, C, D, H, T, L, U, SDS, and J18 (Wang et al., 2004; Shimotohno et al., 2021). CYCD is the subclass with the most members in CYC and mainly regulates the G1 to S phase transition (Potuschak and Doerner, 2001; Menges et al., 2007). The overexpression of *PtoCYCD3* in ‘741’ poplar resulted in larger, wrinkled leaves, a thickened stem, and an increased number of branches (Guan et al., 2021). Auxin appears to influence genes associated with cell division and leaf morphogenesis; for instance, the removal of *IAA28* by miR847 led to the upregulation of both *CYCD3;1* and *CYCB1;1*, which coincided with enhanced auxin signaling. This increase in auxin signaling strengthened meristematic activity and elongated the developmental timeframe (Wang and Guo, 2015). In “84K” poplar, blocking miR393 led to the enhancement of the auxin signaling pathway and the increased expression of *CYCD* and *CYCP* (Chu et al., 2021). In *A. thaliana*, fourteen CDKs have been identified and classified into groups A, B, C, D, E, F, and G (Menges et al., 2005). CYCs bind to specific CDKs to form complexes that phosphorylate proteins, thereby regulating critical events within the cell cycle. The CYCD3;1-CDKA;1 complex in *A. thaliana* improved the cell division capacity of trichomes, whereas the CYCA2;3-CDKB;1 complex exhibited a similar function that was inhibited by the SIM repressor (Wang et al., 2020).

Single-cell sequencing technology has demonstrated remarkable advantages in the study of proliferation and differentiation within the shoot apex meristem and young leaves. Utilizing the shoot apex of approximately 1 m tall wild-type *P. alba* as the study material, morphological observations and structural studies were conducted. The molecular regulatory network governing the development of the shoot apex was elucidated through scRNA-seq, which revealed the various types of cells present in the apical bud. Pseudotime analysis outlined the developmental sequence of different cell organizations. The laboratory has systematically identified the *CYC* and *CDK* gene families, suggesting their involvement in leaf development through leaf transcriptome analysis (He et al., 2022; Liang et al., 2023). In this study, we employed scRNA-seq and bud structure analysis to elucidate the molecular mechanisms by which *CYC* and *CDK* regulate the development of various organizational structures within the apical bud. This research provides novel insights into the roles of the *CYC* and *CDK* gene families in the development of the shoot apex in *P. alba*.

## 2 Materials and methods

### 2.1 Observation of shoot apex structure by scanning electron microscope

We took the shoot apex of a wild-type *P. alba* approximately 1 m in height and carefully removed the larger and more prominent young leaves. The entire bud measure about 1 cm. Samples were prepared using a modified oscillating sectioning method (Zhang et al., 2020). A 3% agarose solution was prepared by heating and dissolving the agarose, then allowing it to cool slightly. The buds were immersed in the agarose, and once it has completely cooled, the agar was trimmed into small pieces. The agar blocks containing the buds was sliced using vibrating sectioning, with the slice thickness set to 40 μm. Slicing should be ceased once the knife reaches the center of the buds. The samples were then immersed in a 2.5% glutaraldehyde solution and placed in a vacuum for 2 h using a vacuum pump. Following this, the samples were washed three times with a 0.1 mol/L PBS solution buffered at pH 7.2, subsequently undergoing a washing process with a gradient ethanol series of 30%, 50%, 70%, 80%, 90%, and 100%. Each ethanol concentration wash lasted for 20 min, during which the samples were gently shaken on ice. The samples were then processed using a carbon dioxide dryer. Place the samples inside, add absolute ethanol, and allow them to dry for approximately 3 h. Ensure that the samples are firmly adhered to conductive tape on the base before proceeding to spray the samples with gold powder. Once these steps are completed, conduct electron scanning tunneling microscope observations and photographic documentation.

### 2.2 Single-cell sequencing of apical bud

At the end of July, the apical buds of 1-year-old white poplar seedlings grown in soil were collected. The length of the shoot apex is approximately 1 cm. Every five plants are mixed as a biological replicate, with a total of two replicates. Protoplast preparation and on-machine sequencing were conducted by GENE DENOVO Biotech. The procedure involved chopping the buds with a blade, followed by digesting the cell wall using digestive enzymes to create a protoplast cell suspension. Subsequently, a cell quality inspection was performed: a small amount of the single-cell suspension was taken, mixed with an equal volume of 0.4% trypan blue dye, and the cells were counted using the Countess II Automated Cell Counter. The concentration of viable cells was adjusted to the ideal range of 1000-2000 cells/μL. Gel beads containing barcoded information were allowed to bind to a mixture of cells and enzymes, which were then introduced into a reservoir and separated by oil, forming gel beads in emulsions (GEMs). Subsequently, the gel beads dissolved to release the capture sequence, which included the barcode sequence, followed by the reverse transcription of the cDNA fragment and sample labeling. The gel beads and oil droplets were then disrupted, and PCR amplification was performed using cDNA as the template. All GEM products were pooled to construct a standard sequencing library. First, the cDNA was fragmented to approximately 200-300 bp, followed by PCR amplification to establish a cDNA library via conventional second-generation sequencing library construction steps, including terminal repair, A-tail addition, and the addition of sequencing junctions P5, P7, and a sample index. Finally, Illumina’s PE150 sequencing mode was employed for high-throughput sequencing of the constructed library.

### 2.3 Raw data quality control

CellRanger (version=7.1, 10x Genomics) software was utilized for preliminary quality control of the sequencing data. The genome of *Populus alba* was from Chinese Academy of Forestry. Seurat objects were created using the R packages Seurat (version=4.0), allowing for the identification of cells with abnormal gene expression (Stuart et al., 2019). The number of gene expressions in normal cells generally remained within a specific range, and the total amount of RNA showed consistency as well. If these two values were excessively high, the cells may belong to multiple cell types. Additionally, high expression of mitochondrial genes often indicates apoptotic cells; this may occur due to adverse effects during protoplast preparation, which could compromise the reflection of the true cellular conditions. Consequently, these cells were eliminated during the screening process. And other R packages including tidyverse, patchwork, ggplot2 and dplyr also involved in the research. The parameters for filtering were as follows: nFeature_RNA > 200 & nFeature_RNA < 7500 & percent.mt < 0.3 & nCount_RNA < 40000. The filtered data were normalized using the NormalizeData function; the global scaling normalization method was applied to normalize the gene expression of each cell based on total expression, multiplied by a scaling factor of 10,000, followed by a logarithmic transformation of the results.

### 2.4 Cell clustering and identification

Using the ScaleData function to scale the data and the RunPCA function for PCA dimensionality reduction reduces the weight of highly variable genes, preventing them from dominating subsequent analyses. Dimensions containing a substantial amount of information were retained, while those with minimal information were eliminated to minimize subsequent data interference. Dimension 20 was selected for PCA reduction. The appropriate resolution argument was determined using the FindNeighbors and FindClusters functions with the R packages dplyr and clustree, selecting a value of 0.7. For cell clustering, Seurat employed the KNN algorithm, and the parameters for running RunUMAP were set as follows: dims = 1:20, n.neighbors = 25, min.dist = 0.1, spread = 2. The dims parameter was set to 20 when running RunTSNE. Indicator information for each cluster was analyzed based on metadata, and the number of genes (nFeature_RNA), total RNA (nCount_RNA), mitochondrial proportion (percent.mt), and cell source sample proportion was calculated for each cluster. Each cluster was distinguished by a unique set of upregulated genes, defined as those expressed in more than 25% of the cells within a cluster. The expression level of this cluster was then compared to that of non-cluster cells. A log2 fold change (log2(FC)) exceeding 0.25 indicated a significant difference in expression levels. Marker genes were identified as criteria for cell clusters and displayed using a bubble plot, as per published literature.

### 2.5 Pseudotime trajectory analysis

For pseudotime analysis, we utilized a subset function to randomly sample 30% of the cells. An object was constructed using the as.CellDataSet function, followed by evaluation of type factors and dispersion, resulting in the selection of high-dispersion genes in Monocle (version=2.28) (Qiu et al., 2017). Dimensionality reduction and cell sequencing were conducted using the reduceDimension and orderCells functions, whereas the differentialGeneTest functions were employed to reconstruct cell development trajectories.

### 2.6 Electrophoretic mobility shift assay

Probes utilized in this study included a 30-bp subfragment (-771 to -741 bp upstream of ATG) derived from the *PoalbCDKA;1* promoter, a 31-bp subfragment (-768 to -737 bp upstream of ATG) from the *PoalbCYCD3;5* promoter, and an additional 30-bp subfragment (-1318 to -1288 bp upstream of ATG) from the *PoalbCYCD3;3* promoter (**Supplementary Table S1**). The EMSA was conducted following the methodology outlined in prior studies (Cai et al., 2023) and adhered to the guidelines provided in the Chemiluminescent EMSA Kit (Beyotime Biotech, Shanghai, China). Briefly, a reaction mixture consisting of 2 μL of protein and 1 μL of the corresponding probe was combined with 2 μL of binding buffer, achieving a total reaction volume of 10 μL. The volume deficiency was adjusted with Nuclease-Free Water, and the mixture was incubated at 25°C for 30 min. Following this, DNA-protein complexes were separated using native polyacrylamide gels containing 4% (w/v) acrylamide and 2.5% (v/v) glycerol. To prevent overheating of the gel, electrophoresis was performed in an ice-water bath. Once electrophoresis was completed, the probe was transferred to a positively charged nylon membrane (Beyotime Biotech, Shanghai, China) utilizing a wet electroporation membrane device in an ice-water bath. After the transfer was completed, the nylon film was placed in a clean plastic dish, positioned 10 cm away from the ultraviolet lamp on the ultra-clean workbench, and subjected to UV cross-linking for 15 min. Following the washing steps with blocking solution, washing solution, and balancing solution, the BeyoECL Moon working solution provided in the kit was employed for development and observation.

## 3 Results

### 3.1 The structure of the shoot apex was observed by scanning electron microscope

**Figure 1:**
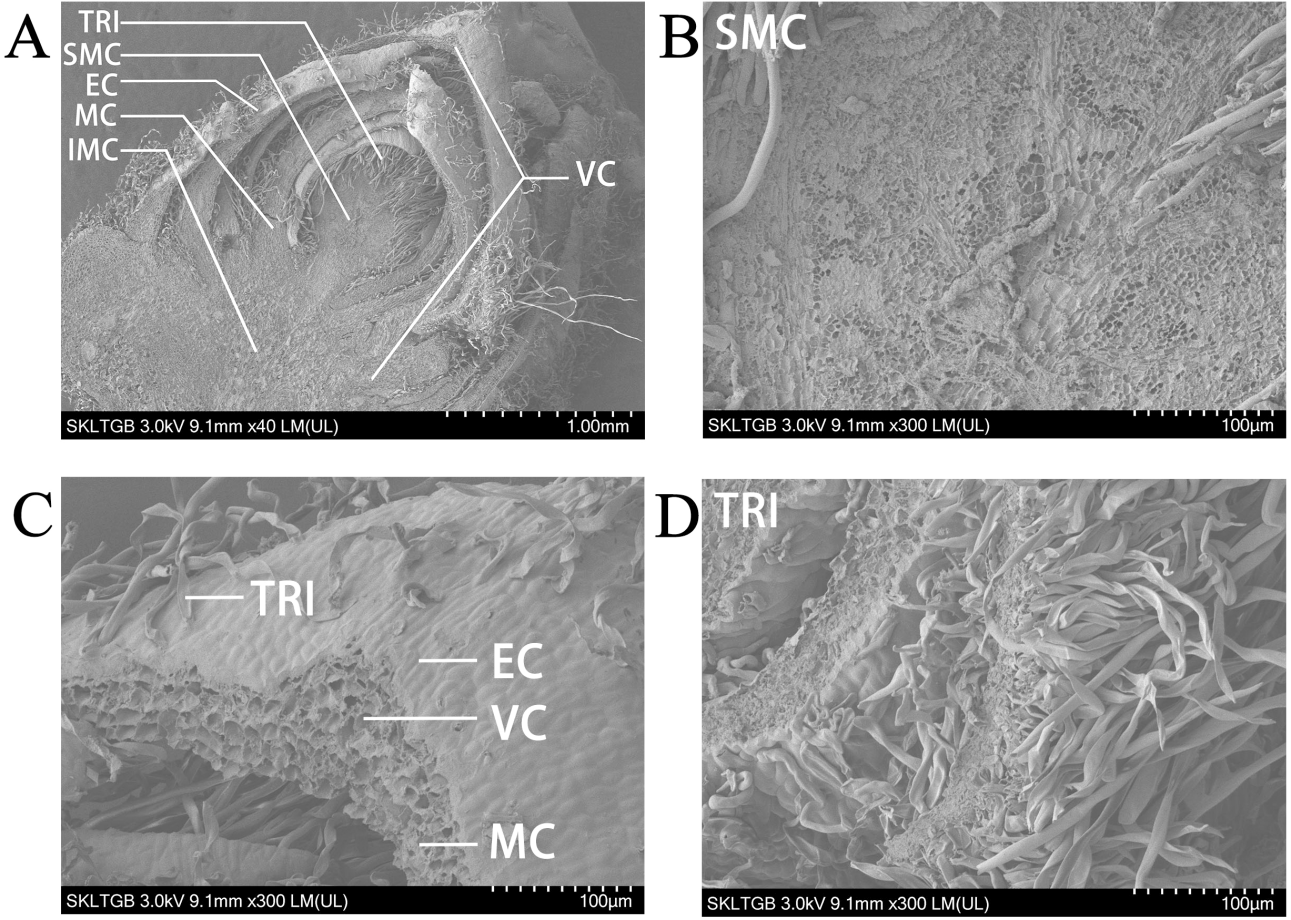
Scanning electron microscope image of shoot apex in *P. alba.* **(A)** Panoramic view of the shoot apex at 40× magnification. **(B)** Shoot apex meristem at 300× magnification. **(C)** Young leaf at 300× magnification. **(D)** Trichomes at 300× magnification. TRI represents trichomes. SMC represents shoot apex meristem cells. EC represents epidermal cells. MC represents mesophyll cells. IMC represents intercalary meristem cells. VC represents vascular cells.

The shoot apex contains leaf primordia and young leaves, making it an ideal subject for precise studies of leaf development. Initially, scanning electron microscopy (SEM) was conducted on the shoot apex of *P. alba*, revealing that the shoot apical meristem occupies a relatively small central portion. Notably, despite the removal of more mature leaves from the outer layers, the shoot apex remains encircled by approximately five layers of young leaves. Gaps between these leaves are populated by numerous trichomes, which are present on certain areas of the leaf surfaces (**Figure 1A**). Upon observing the shoot apical meristem under a 300× electron microscope, it is evident that the cells in this region are very small and densely arranged (**Figure 1B**). At this magnification, four distinct tissue types can be identified in the young leaves. The epidermal cells are tightly arranged to form the epidermis of the young leaves, with trichomes emerging from this layer and taking on a long, strip-like shape. The mesophyll cells exhibit a generally uniform shape without significant differentiation into palisade and spongy tissues, with approximately four cell layers present (**Figure 1C**). Vascular cells are located centrally within the young leaves, surrounded by about nine layers of mesophyll cells with similar diameters. Numerous white trichomes are visible on both the shoot apex and the abaxial surfaces of the leaves of *P. alba*. Under the electron microscope, these trichomes appear as elongated structures and are densely distributed. Most measure around 2 mm in length, whereas some reach approximately 5 mm (**Figure 1D**).

### 3.2 scRNA-seq data filtering and clustering

Utilizing scRNA-seq technology to analyze apical bud cells allows for a precise examination of the gene expression characteristics of individual cells, enabling a comprehensive investigation into the specific roles of CYC and CDK in cell division across various tissues. One-year-old *P. alba* seedlings were selected, and scRNA-seq was conducted on the apical buds (**Supplementary Figure S1**). During the preparation of protoplasts, quality inspection results indicated that the cell counts for the two biological replicates were 1490 cells/μL and 1100 cells/μL, respectively. The total number of cells for each replicate reached 140,000 and 110,000, respectively, with cell viability exceeding 95%. The fragmentation rates recorded were 10% and 8%, respectively (**Supplementary Table S2**). Following processing with Cell Ranger software, bud samples 1 and 2 yielded approximately 510 million and 410 million reads, respectively. The Q30 values for these reads exceeded 90%, with the proportion of effective barcodes exceeding 96% and Q30 values also over 96% (**Supplementary Table S2**). The criteria for retaining cells in this study required that the number of genes be between 200 and 7,500, the proportion of mitochondrial gene expression be less than 30%, and the total RNA amount be less than 40,000. Ultimately, 29,011 cells were screened, with the number of cells in bud 1 and bud 2 being 16,857 and 12,154, respectively, and the total number of aligned genes was 30,167 (**Supplementary Figure S2A**, **Supplementary Table S3**). According to indicator correlation analysis, the number of genes, total RNA amount, and mitochondrial proportion of the two samples were similar. The total RNA amount was inversely proportional to the proportion of mitochondria and directly proportional to the number of genes (**Supplementary Figure S2B**).

After dimensionality reduction, the cells from the two samples exhibited significant overlap, demonstrating strong biological reproducibility (**Supplementary Figure S2C**). 20 prominent principal components were selected as parameters for the subsequent analysis (**Supplementary Figure S2D**). In this study, cluster analysis of cells was performed using a resolution of 0.7 (**Supplementary Figure S2E**) and UMAP and tSNE were employed for nonlinear dimensionality reduction. In the UMAP diagram, cells in 0-16 clusters are separated based on gene expression similarity. The proximity of points indicates higher similarity in the original high-dimensional space. Clusters 0, 1, 2, 4, 5, and 8 are positioned centrally. In contrast, the other clusters demonstrate a greater degree of cell differentiation, facilitating the identification of subsequent cell tissue types (**Supplementary Figure S2F**). The tSNE graph utilizes the similarity between data points in high-dimensional space to create a mapping in low-dimensional space, thereby emphasizing the preservation of local structure (**Supplementary Figure S2G**).

### 3.3 Cluster feature analysis

**Figure 2:**
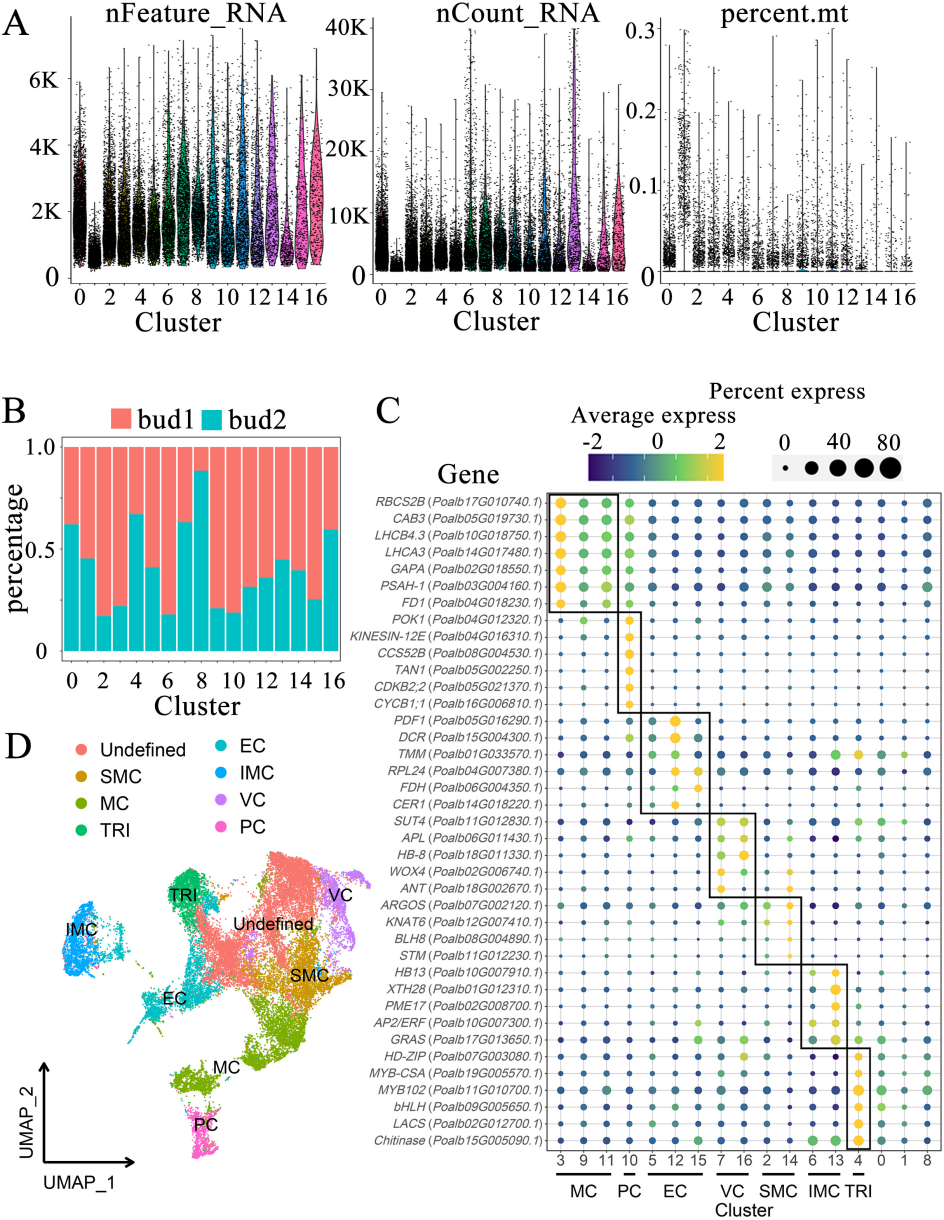
Characterization of cell populations in shoot apex. **(A)** Indicator figure illustrating the gene number (nFeature_RNA), total RNA (nCount_RNA), and proportion of mitochondria (percent.mt) in each cluster. **(B)** Proportions of cell-derived samples within each cluster. **(C)** Expression patterns of marker genes. The color of the bubbles indicates the average gene expression level, with yellow representing higher expression and blue indicating lower expression. The size of the bubbles reflects the cell expression ratio of each gene. The abbreviations MC, PC, EC, VC, SMC, IMC, and TRI denote mesophyll cells, proliferating cells, epidermal cells, vascular cells, shoot apex meristem cells, intercalary meristem cells, and trichomes, respectively. **(D)** Visualization of the cell populations utilizing UMAP.

The indicators for each cluster were thoroughly analyzed, including the number of genes, total RNA quantity, and mitochondrial proportion associated with each cluster (**Figure 2A**). Overall, the indicator information pertinent to most clusters exhibited remarkable similarity. The median number of genes ranged from 2000 to 5000, with the highest median count of 7800 observed in cluster 13 and the lowest recorded in cluster 1 at 874. The median total RNA amount of most clusters remained at 1300-2200, with cluster 1 and cluster 14 being lower at 597 and 913, respectively (**Supplementary Table S5**). Furthermore, the proportion of mitochondrial gene expression across most cells approached 0. By evaluating the proportion of cell-derived samples within each cluster, we discerned the sample specificity of certain clusters (**Figure 2B**). In the overall cell composition, sample 1 accounted for 58.11% of the cells, whereas sample 2 comprised 41.89%. Notably, the proportion of sample 1 in clusters 2, 3, 6, 9, and 10 was significantly higher, reaching 83%, 78%, 82%, 79%, and 81%, respectively; however, the proportion of sample 1 in cluster 8 was considerably low at only 12%. The cell counts in clusters 0, 1, and 2 were elevated, with respective tallies of 4052, 3409, and 3279, whereas clusters 13, 14, 15, and 16 exhibited lower cell counts of 649, 427, 391, and 358, respectively (**Supplementary Table S5**). Generally, the number of upregulated genes in each cluster exceeded 300; however, some clusters displayed fewer upregulated genes, including 219 in cluster 1, 224 in cluster 6, and 176 in cluster 14 (**Supplementary Table S4**).

### 3.4 Identification of tissue groups

According to the marker genes of each tissue group in the shoot apex, we labeled each cluster accordingly (**Figure 2C**, **Supplementary Table S7**). Clusters 3, 9, and 11 were designated as mesophyll cells (MC). This study selected several genes associated with chlorophyll and photosynthesis as marker genes, including *RBCS2B* (*Poalb17G010740.1*), which plays a role in carbon dioxide fixation (Zhang et al., 2021), as well as *CAB3* (*Poalb05G019730.1*), *LHCB4.3* (*Poalb10G018750.1*), and *LHCA3* (*Poalb14G017480.1*), which are involved in light harvesting (Kim et al., 2021). Furthermore, the genes glyceraldehyde-3-phosphate dehydrogenase (*GAPA*) (*Poalb02G018550.1*), *PSAH-1* (*Poalb03G004160.1*) within the photosystem, and *FD1* (*Poalb04G018230.1*), related to electron transfer, were also identified. Cluster 10 comprises dividing cells, referred to as proliferating cells (PC). The marker genes associated with this cluster include *POK1* (*Poalb04G012320.1*) and *KINESIN-12E* (*Poalb04G016310.1*), which facilitate vascular movement, as well as the ubiquitination-related gene *CCS52B* (*Poalb08G004530.1*) (Tian et al., 2019), along with *TAN1* (*Poalb05G002250.1*) (Conde et al., 2022), *CDKB2;2* (*Poalb05G021370.1*), and *CYCB1;1* (*Poalb16G006810.1*) (Liang et al., 2023). Clusters 5, 12, and 15 were identified as epidermal cell (EC) clusters. The marker genes associated with these clusters include *PDF1* (*Poalb05G016290.1*), acyltransferase *DCR* (*Poalb15G004300.1*), and *TMM* (*Poalb01G033570.1*), which is involved in stomatal development, along with *RPL24* (*Poalb04G007380.1*), the fatty acid synthesis-related gene *FDH* (*Poalb06G004350.1*), and *CER1* (*Poalb14G018220.1*) (Zhang et al., 2021). The vascular cell (VC) group includes clusters 7 and 16. The marker genes associated with this group feature phloem- and xylem-related genes such as the companion cell-related *ANT* (*Poalb18G002670.1*), *WOX4* (*Poalb02G006740.1*), the xylem-associated *HB-8* (*Poalb18G011330.1*), and the phloem-related *APL* (*Poalb06G011430.1*) (Zhang et al., 2021), as well as *SUT4* (*Poalb11G012830.1*) (Conde et al., 2022). The marker genes for shoot apex meristem (SMC) cells include the shoot apex meristem genes *STM* (*Poalb11G012230.1*), *BLH8* (*Poalb08G004890.1*) (Conde et al., 2022), *KNAT6* (*Poalb12G007410.1*), and the auxin-regulated organ size gene *ARGOS* (*Poalb07G002120.1*) (Zhang et al., 2021). Although some marker genes did not satisfy the criteria for up-regulated genes, they demonstrated high expression levels in clusters 2 and 14 compared to other clusters. Consequently, these clusters were identified as shoot apical meristems. The intercalary meristem cells (IMC) include clusters 6 and 13, located at the base of the shoot apex and internode. They are primarily responsible for cell elongation, expansion, and differentiation, contributing to cell wall softening. Marker genes for this tissue type respond to ethylene or gibberellin and include *HB13* (*Poalb10G007910.1*), glycan hydrolase *XTH28* (*Poalb01G012310.1*), pectinesterase *PME17* (*Poalb02G008700.1*), the ethylene-responsive gene *AP2*/*ERF* (*Poalb10G007300.1*), and the gibberellin-responsive *GRAS* (*Poalb17G013650.1*). The stem tips and young leaves of *P. alba* are densely covered with trichomes. This study identified cluster 4 as trichomes (TRI), which express the chitinase gene *Chitinase* (*Poalb15G005090.1*) to withstand external stresses such as fungal attacks. Additionally, glycerol-phosphate acyltransferase *LACS* (*Poalb02G012700.1*) is involved in cutin synthesis (Berhin et al., 2021). Several transcription factors that promote trichome growth were also identified, including *MYB102* (*Poalb11G010700.1*), *MYB-CSA* (*Poalb19G005570.1*), *bHLH* (*Poalb09G005650.1*), and *HD-ZIP* (*Poalb07G003080.1*) (Chalvin et al., 2020).

Three clusters, labeled as 0, 1, and 8, cannot be distinctly identified based on marker genes. These clusters contain cell counts of 4,052, 3,409, and 1,523, respectively, together representing 30.9% of the total cell population (**Supplementary Table S5**). Notably, the TRI marker genes exhibit a certain level of expression in cluster 0. The GO enrichment reveals that the Cluster 0 focus on fungus defense, salt response and detection of chemical stimulus, which shows the comparability with Cluster 4 (**Supplementary Table S6**). These evidences indicate a degree of similarity between clusters 0 and 4 (**Figure 2C**). The GO enrichment of Cluster 1 concentrates on amino acid modification, transcription regulation, chromatin remodeling and calcium ion transport. Cluster 8 may involve in the biological and abiotic stress according to the GO enrichment.

Following the identification of cell types, the UMAP map was re-annotated and organized into eight major groups (**Figure 2D**). Undefined groups are positioned centrally, whereas other groups are distributed around the UMAP map (**Figure 2D**), suggesting a high degree of differentiation among these groups. Some groups are relatively close to one another, hinting at potential similarities in gene expression between paired groups.

### 3.5 Pseudotime trajectory analysis

**Figure 3:**
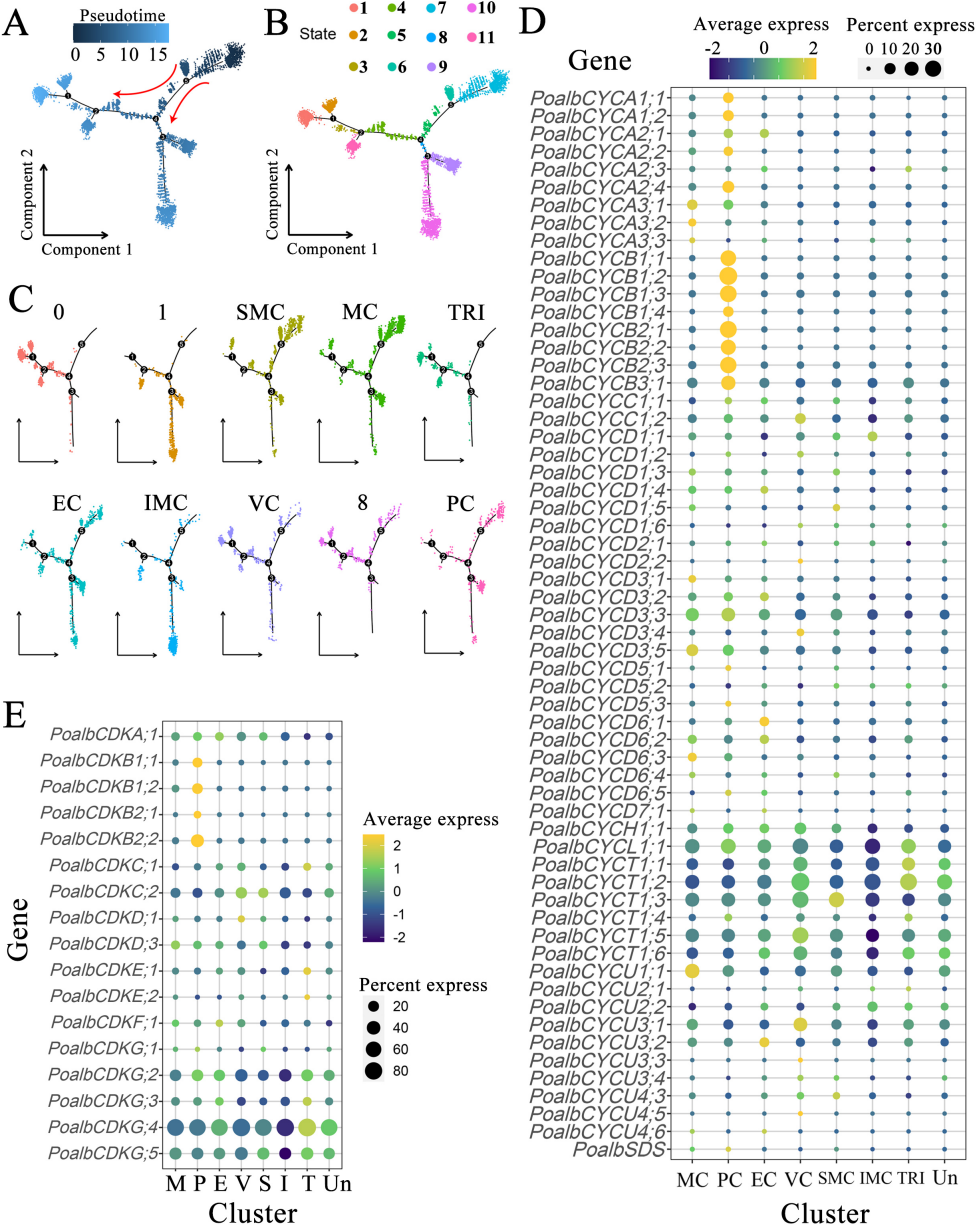
Pseudotime analysis and expression pattern of *CYC* and *CDK* gene families. **(A)** Pseudotime analysis is conducted based on pseudotime values. In a two-dimensional spatial trajectory, each point corresponds to a cell. The numbers within the black circles denote nodes that represent distinct cellular states along the trajectory. The depth of color and the red arrows indicate the progression order of pseudotime. **(B)** Pseudotime analysis according to cell states, with colors representing different cell states. **(C)** Pseudotime analysis of cell populations. **(D)** Expression pattern of *CYC* genes. **(E)** Expression pattern of *CDK* genes. The color of the bubbles indicates the average gene expression level, with yellow representing higher expression and blue indicating lower expression. The size of the bubbles reflects the cell expression ratio of each gene. The abbreviations MC (M), PC (P), EC **(E)**, VC (V), SMC (S), IMC **(I)**, TRI (T), and Un denote mesophyll cells, proliferating cells, epidermal cells, vascular cells, shoot apex meristem cells, intercalary meristem cells, trichomes, and undefined groups, respectively.

Pseudotime trajectory analysis facilitates the evaluation of cell distribution in both time and space. It visualizes the placement of each cell group along the trunk cell, constructs the developmental processes of cell lineages, and assesses the transitions in gene expression across different cell groups (**Figure 3A**). During cellular development, the cell locus is categorized into three primary branches, including a total of eleven states (**Figure 3B**). According to cell type annotation results, the true starting point of the pseudotime trajectory extends from right to left, with SMC and MC emerging first. These cells begin to diverge into two major branches at node 4 (**Figure 3C**). The trajectory distribution of PC parallels that of shoot apical meristem and mesophyll cells. VC appear in relatively smaller numbers at the initial stage but are predominantly concentrated within the substantial trajectory of node 4 that diverges to the left. Nearly all cells in the TRI are located in the middle and later sections of the left track, indicating that this tissue undergoes differentiation at a later stage. Similarly, the IMC shows a low cell count at the initial phase of the trajectory, although most of the cells are located towards the end of the downward trajectory at node 4, suggesting late differentiation for this tissue as well. The distribution of EC does not exhibit significant enrichment, with cells observed at the beginning, middle, and end of the overall trajectory, indicating that the differentiation of epidermal cells occurs throughout the entire developmental process.

Clusters 0, 1, and 8 lacked distinct cell type annotations (**Figure 3C**). In the pseudotime analysis, cells in cluster 0 were predominantly located in the posterior region of the left trajectory and may share a similar gene expression profile with trichomes. Cluster 1 was primarily found in the downward branch. Cells in Cluster 8 exhibited a distribution across the anterior, middle, and posterior regions of the locus. Overall, the pseudotime analysis indicated that in the shoot, apical meristem cells and mesophyll cells emerged first, followed by the differentiation of vascular cells and intercalary meristem cells, which gradually matured over time. The differentiation of trichomes occurred last, whereas the development of epidermal cells accompanied the entire developmental process.

### 3.6 Specific expression of *CYC*, *CDK*, and auxin-related genes

The shoot apex consists of leaf primordia, young leaves, and shoot apex meristems, where cells are actively dividing. Utilizing scRNA-seq to examine the expression of the CYC and CDK gene families in various leaf groups is crucial for understanding leaf polarity establishment, growth, and development. Within the CYC family, *CYCA* exhibited prominent expression in mesophyll and proliferating cells, indicating its involvement in mesophyll cell division processes. In contrast, *CYCA2;1* showed high expression levels in the epidermis. All members of the *CYCB* family were predominantly found in proliferating cells. Interestingly, several marker genes associated with photosynthesis were also highly expressed in dividing groups, suggesting that this cluster may comprise mesophyll cells in a mitotic state. It is plausible that *CYCB* shares functions similar to *CYCA*, particularly concerning mesophyll cell division. The *CYCD* subfamily contains the most members of the CYC family, with each member exhibiting significant variation in expression patterns across different tissues. Notably, five members of the *CYCD3* subgroup were highly expressed in mesophyll cells, epidermal cells, and shoot apical meristem cells. Members *CYCD6;1* and *CYCD6;2* had elevated expression levels in vascular tissues, whereas *CYCD6;3* was primarily localized in mesophyll cells. Members of the *CYCH*, *CYCL*, and *CYCT* subclasses were uniformly expressed across all tissues of the apical bud, with relatively consistent expression levels. This suggests that these subfamilies may play a widespread role in the cell division processes of various apical bud tissues. *CYCU1;1* was predominantly found in mesophyll cells, *CYCU3;1* primarily localized in vascular cells, and *CYCU3;2* was specifically concentrated in epidermal cells. This distribution indicates that different members of this subclass may fulfill distinct roles in the morphogenesis of various leaf tissues (**Figure 3D**, **Supplementary Table S4**).

In the CDK gene family, *CDKA;1* was expressed in both leaf organs and the SAM. All four members of the *CDKB* subgroup were predominantly expressed in proliferating cells. Member *CDKC;2* was notably expressed in vascular cells and the SAM, whereas *CDKF;1* demonstrated abundant expression in epidermal cells. Members *CDKG;2*, *CDKG;4*, and *CDKG;5* were expressed across a variety of identified tissues, with their expression levels particularly concentrated in trichomes and unidentified clusters (**Figure 3E**, **Supplementary Table S4**).

**Figure 4:**
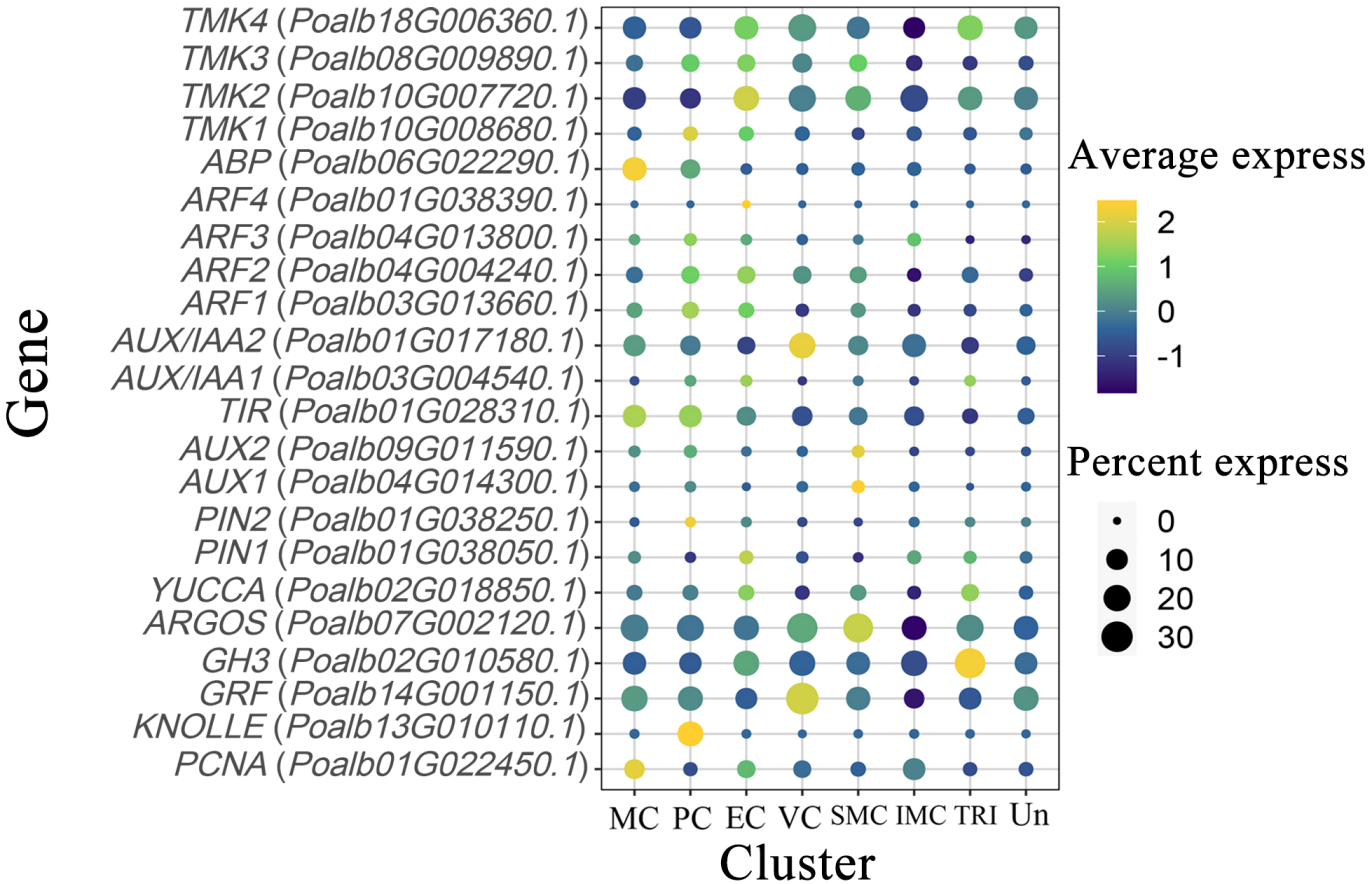
Expression pattern of auxin-related genes. The color of the bubbles indicates the average gene expression level, with yellow representing higher expression and blue indicating lower expression. The size of the bubbles reflects the cell expression ratio of each gene. The abbreviations MC, PC, EC, VC, SMC, IMC, TRI, and Un denote mesophyll cells, proliferating cells, epidermal cells, vascular cells, shoot apex meristem cells, intercalary meristem cells, trichomes, and undefined groups, respectively.

TMK kinase is predominantly expressed in proliferating cells and epidermal cells, and it is also present in vascular and shoot apex tissues. The ABP protein, which interacts with TMK kinase, is enriched and expressed in mesophyll cells, with some expression observed in dividing cells. ARF transcription factors are expressed to varying extents in mesophyll, dividing, and epidermal cells. *AUX*/*IAA2* is concentrated in vascular tissues, whereas *AUX*/*IAA1* is found in dividing and epidermal cells. The expression pattern of *TIR* genes is similar to that of ABP. Two *AUX* genes are highly expressed in the SAM. *PIN2* and *KNOLLE* are predominantly expressed in proliferating cells, whereas *PIN1* and *YUCCA* are expressed in both epidermal and trichome cells. The *ARGOS* gene is primarily expressed in the SAM and vascular cells. *PCNA* shows high expression levels in the mesophyll, whereas *GRF* is notably expressed in vascular tissues. The *GH3* gene, known for promoting auxin degradation, is highly expressed in trichomes (**Figure 4**, **Supplementary Table S4**).

### 3.7 EMSA

**Figure 5:**
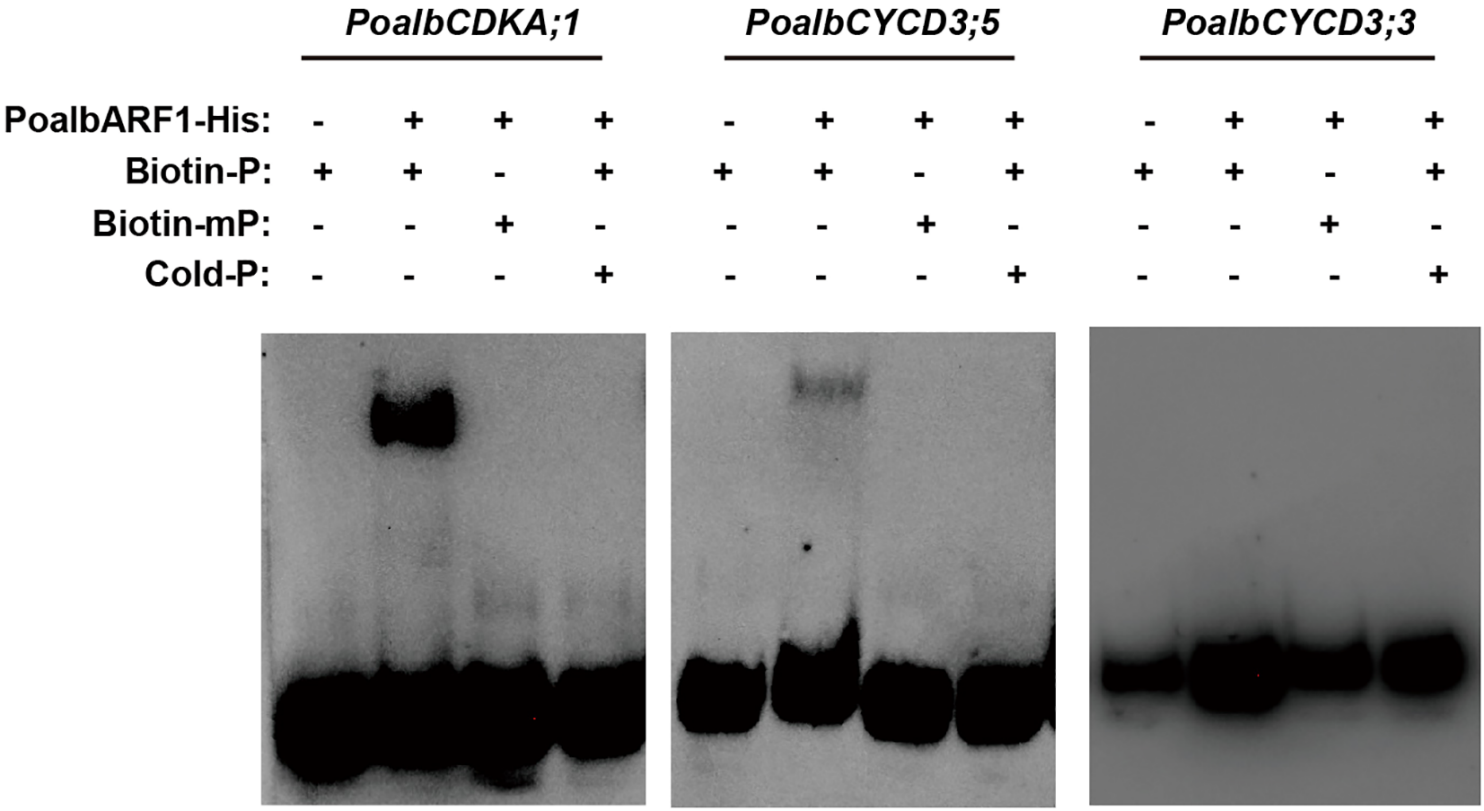
PoalbARF1 directly binds to the promoter of *PoalbCDKA;1* and *PoalbCYCD3;5.* Electrophoretic mobility shift assay (EMSA) revealed this direct binding of PoalbARF1 to the promoters of *PoalbCDKA;1* and *PoalbCYCD3;5in vitro*. Biotin-P denotes biotin-labeled probes; Biotin-mP, biotin-labeled mutation probes; Cold-P, biotin-unlabeled probes. The symbols + and – indicate the presence and absence of the corresponding probes or proteins, respectively.

Previous studies indicated that CYC and CDK exhibit a strong correlation with the auxin signaling pathway in leaf development (Liang et al., 2023). It was speculated that the ARF transcription factor may play a crucial role in activating the transcription of *CYC* and *CDK*. To investigate this association, we conducted EMSA experiments to examine the interactions between PoalbARF1 and the promoters of *PoalbCDKA;1*, *PoalbCYCD3;3*, and *PoalbCYCD3;5*. The EMSA results demonstrated that PoalbARF1 can directly bind to the promoters of *PoalbCDKA;1* and *PoalbCYCD3;5in vitro*; however, it does not bind to the promoter of *PoalbCYCD3;3* (**Figure 5**).

## 4 Discussion

### 4.1 ScRNA-seq analysis revealed the distribution of cells in the shoot apex

The development of the shoot apex is governed by a complex gene regulatory network. Investigating this developmental process can provide valuable insights into the mechanisms by which plants exert precise control over their growth and morphogenesis through the regulation of gene expression. In this context, SEM has been employed to examine the ultrastructure of the shoot apical meristem and young leaf epidermal cells within the apical bud of poplar trees. Observations revealed that these cells are small in size and densely packed. Notably, the mesophyll tissue exhibited a lack of clear differentiation into palisade and spongy layers, with only approximately four cell layers present. In contrast, vascular tissues were characterized by a significantly higher number of layers, around nine, and displayed diameters comparable to those of the mesophyll cells. Trichomes emerged from the epidermis in a strip-like formation, growing densely and varying in length; the majority measured around 2 mm, although some trichomes reached lengths of up to 5 mm (**Figure 1**). Recent advancements in scRNA-seq technology within botanical research have highlighted its considerable potential to elucidate cellular heterogeneity, cell differentiation trajectories, and mechanisms of response to environmental changes. Following the filtering of the scRNA-seq data from *P. alba* apical buds, a total of 29,011 cells were identified, with 30,167 genes compared (**Supplementary Table S3**). The cells were organized into 17 distinct clusters. With the exception of cluster 1, most cluster data appear to be normal. The median number of genes ranges from 2,000 to 5,000, whereas the median total RNA amounts range between 1,300 and 2,200. In most cells, the proportion of mitochondrial genes is approximately 0% (**Supplementary Figure S2E**, **Supplementary Table S5**). The overall quality of the data is substantial. The basic structure of the shoot apex of *P. alba* was obtained through SEM, and the seven main tissue cell types of the shoot apex were identified through scRNA-seq and marker genes. Pseudotime analysis demonstrated that shoot apex meristem cells and mesophyll cells appeared first, followed by the differentiation of vascular cells and intercalary meristem cells, which gradually matured over time; trichoid cells differentiated last, with epidermal cell differentiation occurring throughout the entire developmental process. Clusters 0, 1, and 8 did not annotate specific cell groups, but pseudotime trajectory analysis indicated that cluster 0 cells exhibited gene expression patterns similar to those of trichomes.

### 4.2 The *CYC* and *CDK* gene families specifically expressed in the different organizations of apical bud

The expression patterns of cell cycle-related genes in various tissues were observed as follows: *CYCA*, *CYCB*, *CYCD3*, *CYCD6;3*, *CYCU1;1*, and *CDKB* exhibited predominantly high expression in mesophyll cells; *CYCD6;1*, *CYCD6;2*, and *CYCU3;1* were primarily expressed in vascular cells; *CDKF;1* displayed concentrated expression in epidermal cells; and *CDKC;2*, along with *CYCD3*, showed pronounced expression in stem tip meristem cells. Moreover, *CYCH*, *CYCL*, *CYCT*, *CDKA;1*, *CDKG;2*, *CDKG;4*, and *CDKG;5* were generally expressed across all tissues of the shoot apex (**Figure 3D**, **Supplementary Table S4**), indicating their potential involvement in cell division processes across various tissue types. Because some marker genes involved in photosynthesis are also well expressed in the dividing group, this group may contain part of mesophyll cells in the state of division. The pseudotime analysis demonstrated a significant overlap in the trajectory distribution of proliferating cells with that of mesophyll cells, further supporting this inference. Therefore, in conjunction with predictions from single-cell analyses, it can be concluded that *CYCA*, *CYCB*, *CYCD3*, and *CDKB* collectively regulate the division of mesophyll cells. *CYCD6;1*, *CYCD6;2*, *CYCU3;1*, and *CDKC;2* are implicated in the division of vascular cells, whereas *CYCD3*, *CYCU3;2*, and *CDKF;1* are involved in epidermal cell division. Additionally, *CYCD3* and *CDKC;2* contribute to the division of stem apex meristem cells, and *CYCH*, *CYCL*, *CYCT*, *CDKA;1*, *CDKG;2*, *CDKG;4*, and *CDKG;5* are broadly involved in cell division across various tissues. CYC proteins regulate cell division and mediate plant cell responses to external signals during the G1 phase, whereas members of the CYCD3 group facilitate the transition into the S phase in response to plant hormones and spatial signals (Riou-Khamlichi et al., 1999). Prolonged expression of *CYCD3;1* is a critical factor in initiating plant cell expansion and differentiation (Chua et al., 2002; Schruff et al., 2006). The application of scRNA-seq technology in the study of plant shoot apex development reveals dynamic gene expression changes during shoot apex and young leaf development, allowing for a more precise analysis of intercellular heterogeneity and functional differentiation.

### 4.3 Auxin-related genes regulate the expression of CYC and CDK to promote cell proliferation

The regulatory mechanisms of the auxin signaling pathway are highly complex. At its core, the pathway involves TIR1/AFB (Transport Inhibitor Resistant 1/Auxin Signaling F-Box) functioning as the substrate recognition subunit of the SCF (SKP/CULLIN1/F-Box) E3 ubiquitin ligase, along with the interaction and subsequent ubiquitination of AUX/IAA proteins (Gray et al., 1999, Gray et al., 2001; Qi et al., 2022). Auxin initiates the ubiquitination and degradation of AUX/IAA proteins by binding to TIR1/AFB, consequently influencing the expression of downstream genes that regulate plant growth and development (Dharmasiri et al., 2005; Kepinski and Leyser, 2005). In mesophyll cells, TMK kinase, ABP, TIR, ARF, AUX/IAA1, PIN2, KNOLLE, and PCNA may all play their respective roles. In epidermal cells, TMK kinase, *ARF*, and *AUX*/*IAA1* are well expressed. In vascular cells, *AUX*/*IAA2* and *GRF* may regulate their development. Notably, in the shoot apical meristem, higher expression levels of *AUX* and *ARGOS* are observed. Our laboratory’s previous investigations revealed a strong correlation between the auxin signaling pathway and CYC and CDK in leaf development, as assessed through WGCNA (Liang et al., 2023). Results from scRNA-seq not only confirmed this hypothesis but also expanded and refined it. In mesophyll cells, the pronounced expression of *PCNA* and *KNOLLE* indicates that these cells are in a state of active division. Moreover, TMK kinase and ABP form a complex that interacts with auxin to facilitate the efflux of hydrogen ions, thereby acidifying the cell wall (Lin et al., 2021). PIN2 aids in the efflux of auxin, promoting the release of ARF transcription factors from the AUX/IAA-TIR complex (Barbosa et al., 2018; Forestan and Varotto, 2012; Nishimura et al., 2009). By binding to the promoter, these transcription factors enhance the expression of *CYCA*, *CYCB*, *CYCD*, and *CDKB*, thereby facilitating mesophyll cell division (Shimotohno et al., 2021; Himanen et al., 2002, Himanen et al., 2004; Okushima et al., 2014; Sanz et al., 2011). At the transcriptional level, upstream transcription factors regulate the expression of the *PIN* gene in response to endogenous and exogenous auxin signals. For example, *ARF7* and *FLP* transcription factors collaborate to regulate the transcription of *PIN3*, mediating the initial stages of lateral root formation (Chen et al., 2015). These auxin-related genes directly or indirectly influence CYC and CDK, thereby affecting the cell division processes in related tissues. Furthermore, we employed EMSA to investigate the regulatory effects of ARF on the *CDKA;1* and *CYCD3* genes (**Figure 5**). The results revealed that ARF1 can directly regulate their promoters. It showed a possible way to find the new regulatory factors in plant development by scRNA-seq. Additionally, more experiments, including luciferase assay, yeast hybridization, and transgenic technology, should be employed to prove the connection of auxin signal and CYC and CDK in leaf development.

### 4.4 The cell cluster differentiation process inferred from scRNA-seq needs further verification

The gene *GH3* functions to promote auxin degradation, and its elevated expression in trichomes suggests that the development of these structures may be independent of auxin stimulation. Trichomes can be composed of multicellular, cuticular coating, and their main function is to resist external pests and bacteria, as well as to prevent the loss of water (Berhin et al., 2021). Furthermore, *CYCB* is recognized as a crucial gene involved in the division of trichome cells (Chalvin et al., 2020). However, no enriched expression of *CYCB* was found in trichome cells, and it is possible that the dividing trichome cells were divided into proliferating cells during cell clustering. Utilizing scRNA-seq technology enables the precise clustering of proliferating cells into distinct subgroups. This approach can effectively classify proliferating cells according to their tissue of origin, thereby facilitating a more refined investigation into the roles of CYC and CDK in the cell division processes across different tissues.

The combination of scRNA-seq, bulk RNA-seq, and spatial transcriptome sequencing technology can further cluster and distinguish subgroups of each tissue and may classify specific cells belonging to each tissue, such as palisade and sponge cells with low differentiation in mesophyll tissue, cells partly related to stomatal differentiation in epidermal tissue, and paraxial and apaxial epidermal cells. This will help refine the various tissue types and may find that CYC and CDK are involved in the process of cell division in various elaborate tissues. Additionally, plant cells frequently communicate with each other through cytokines, membrane proteins, and plasmodesmata. By combining scRNA-seq technology with the construction of developmental models, we can analyze intercellular communication and statistically infer interactions among different cell types based on their respective receptors and ligands. This integrative approach fosters a deeper understanding of the intercellular communication networks and the dynamic signaling processes that govern cellular interactions during development.

## 5 Conclusion

The cell groups of the shoot apex are divided into mesophyll cells, proliferating cells, epidermal cells, vascular cells, shoot apical meristem cells, intercalary meristem cells, and trichomes. Shoot apex meristem cells and mesophyll cells appeared first, then vascular cells and intercalary meristem cells began to differentiate and gradually matured with the passage of time. The trichomes differentiated last, and epidermal cell differentiation accompanied the whole development process. *CYC* and *CDK* specifically involved in the different organization of apical buds. Auxin signaling pathway genes were highly correlated with *CYC* and *CDK* gene families in the shoot apex and young leaf development. The ARF1 directly combined the promoters of *CDKA;1* and *CYCD3;5*. In this study, we utilized scRNA-seq to delineate the sequential differentiation blueprint of shoot apex cell populations and to construct the molecular framework underlying the role of *CYC* and *CDK* gene family members in regulating the proliferation of shoot apex cells in *P. alba*.

## Data Availability

The datasets presented in this study can be found in online repositories. The names of the repository/repositories and accession number(s) can be found in the article/**Supplementary Material**.
