# Supplementary material for: Single-cell RNA sequencing of shoot apex reveals the mechanism of cyclin regulating cell division via auxin signaling pathway in Populus alba
Source: Front Plant Sci. 2025 Mar 4;16:1555388. doi: 10.3389/fpls.2025.1555388 (PMC11913855; doi:10.3389/fpls.2025.1555388)
Supplement: Supplementary file 1 [file DataSheet1.zip › Supplementary material/Supplementary_Figures.docx]

Supplementary Material

## Supplementary Figure


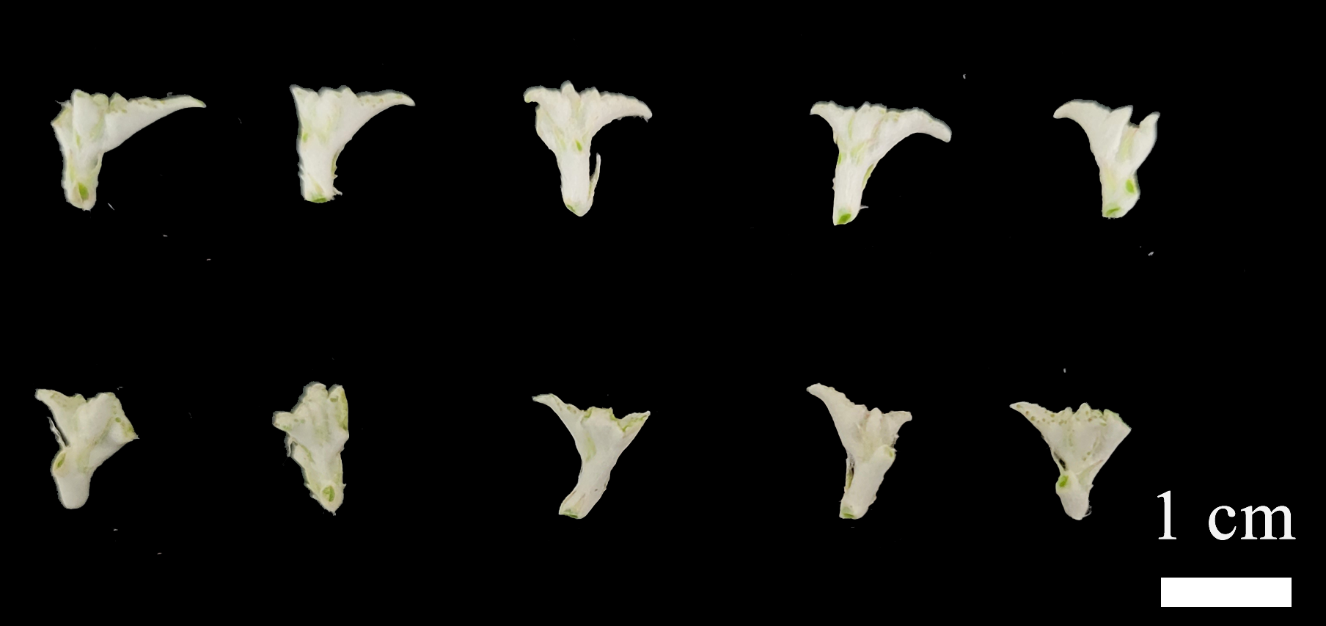


**Supplementary Figure 1.** The bud using for scRNA-seq


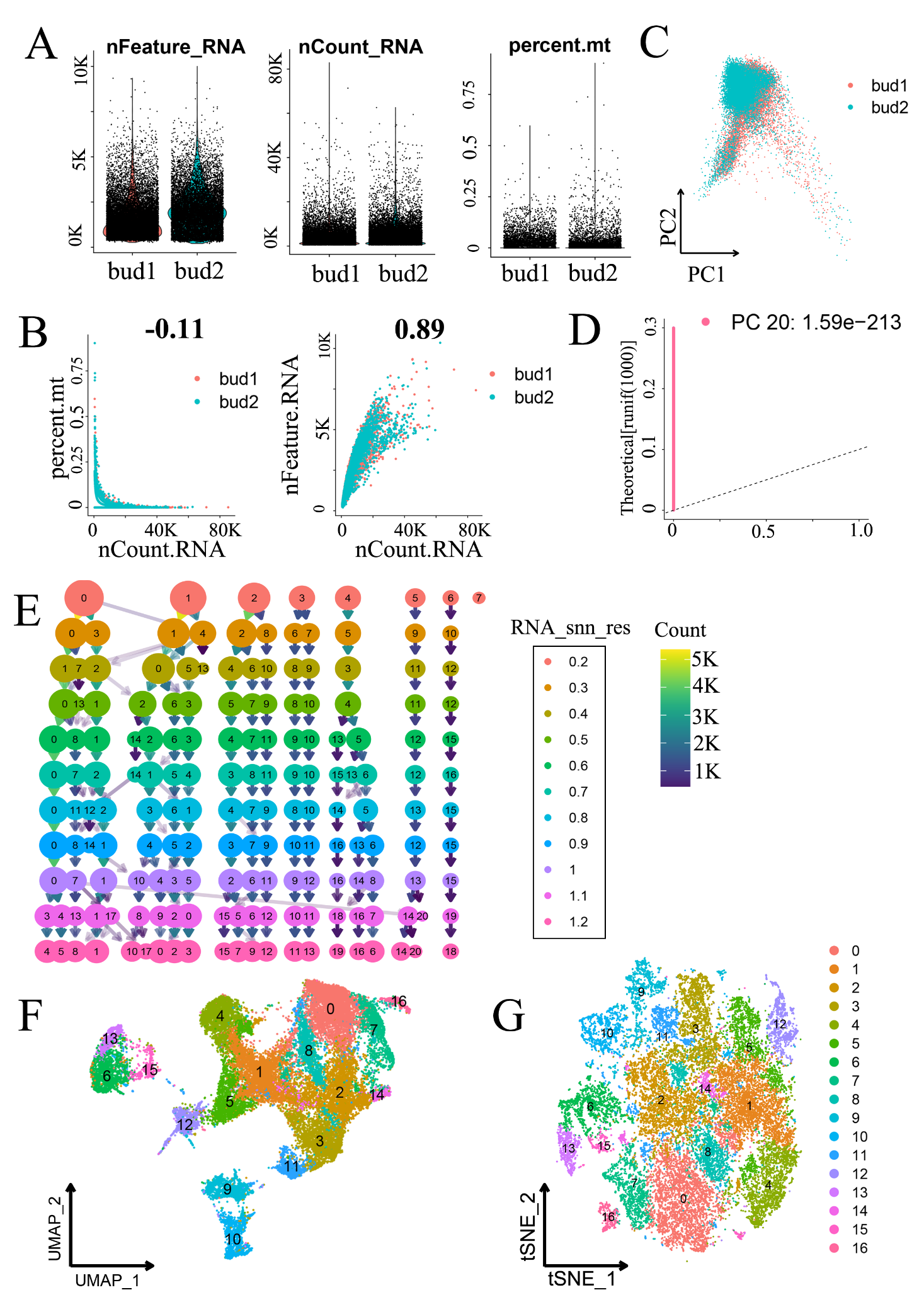


**Supplementary Figure 2. Single-cell clustering analysis of shoot apex in *P. alba***

(A) Indicator figure of the gene number (nFeature_RNA), total RNA (nCount_RNA), and proportion of mitochondria (percent.mt) in samples. (B) The relationship of nCount_RNA and percent.mt and the relationship of nCount_RNA and nFeature_RNA. (C) PCA dimensionality reduction of cells. (D) The selection of dimension. (E) Screening of resolution. (F) Visualization of the 17 cell clusters using UMAP. Dots represent individual cells. Number = 29011. (G) Visualization of the 17 cell clusters using tSNE.
